# Molecular Characterization of Cold Adaptation of Membrane Proteins in the *Vibrionaceae* Core-Genome

**DOI:** 10.1371/journal.pone.0051761

**Published:** 2012-12-17

**Authors:** Tim Kahlke, Steinar Thorvaldsen

**Affiliations:** 1 Department of Chemistry, Faculty of Science and Technology, University of Tromsø, Tromsø, Norway; 2 Department of Mathematics and Statistics, Faculty of Science and Technology, University of Tromsø, Tromsø, Norway; 3 The Norwegian Structural Biology Centre, University of Tromsø, Tromsø, Norway; J. Craig Venter Institute, United States of America

## Abstract

Cold-adaptation strategies have been studied in multiple psychrophilic organisms, especially for psychrophilic enzymes. Decreased enzyme activity caused by low temperatures as well as a higher viscosity of the aqueous environment require certain adaptations to the metabolic machinery of the cell. In addition to this, low temperature has deleterious effects on the lipid bilayer of bacterial membranes and therefore might also affect the embedded membrane proteins. Little is known about the adaptation of membrane proteins to stresses of the cold. In this study we investigate a set of 66 membrane proteins from the core genome of the bacterial family *Vibrionaceae* to identify general characteristics that discern psychrophilic and mesophilic membrane proteins. Bioinformatical and statistical methods were used to analyze the alignments of the three temperature groups mesophilic, intermediate and psychrophilic. Surprisingly, our results show little or no adaptation to low temperature for those parts of the proteins that are predicted to be inside the membrane. However, changes in amino acid composition and hydrophobicity are found for complete sequences and sequence parts outside the lipid bilayer. Among others, the results presented here indicate a preference for helix-breaking and destabilizing amino acids Ile, Asp and Thr and an avoidance of the helix-forming amino acid Ala in the amino acid composition of psychrophilic membrane proteins. Furthermore, we identified a lower overall hydrophobicity of psychrophilic membrane proteins in comparison to their mesophilic homologs. These results support the stability-flexibility hypothesis and link the cold-adaptation strategies of membrane proteins to those of loop regions of psychrophilic enzymes.

## Introduction

Cold adapted bacteria colonize habitats that are hostile to most organisms, e.g. the arctic ocean or the deep-sea, with temperature minima close to or even below the freezing point of water. To maintain growth and survival at these low temperatures cold-adapted (psychrophilic) bacteria require an array of specific adaptations in the cellular components, protein synthesis machinery and enzymes [1], [2]. The main challenges psychrophiles have to overcome are a decrease in enzyme activity and increased viscosity of the aqueous environment due to the low temperature. In terms of enzyme activity, significant differences in amino acid composition and three-dimensional structure of cold-adapted enzymes have been reported in comparison to their mesophilic counterparts [3]–[6]. In general, the results show that psychrophilic enzymes tend to be more flexible and less stable to maintain their catalytic activity. However, studies on psychrophilic enzymes produced differing results, indicating that psychrophilic organisms developed more than one strategy to adapt to the cold [7], [8].

Another crucial aspect in cold-adaptation is the deleterious effect of low temperature on the lipid membrane of bacteria. A lipid bilayer, with a strongly hydrophobic interior, is the structural base for all biological membranes and the surrounding environment for most membrane proteins. As temperature decreases, lipids lose their fluidity and ultimately pass through a transition to form a gel phase, in which the molecules are packed more tightly and motion is highly reduced. This eventually leads to a loss of function of the membrane itself as well as of the proteins that are embedded in or interact with the lipid bilayer. In order to maintain membrane fluidity psychrophilic bacteria show an altered lipid composition with increased ratio of unsaturated fatty acyl residues, cis double bonds, chain shortening, and methyl branching. These changes are mediated through modification of pre-existing lipids by cold-shock-activated enzymes and by de novo synthesis of specific enzymes [9], [10]. However, much less is known about the corresponding changes in membrane proteins in response to the low temperature. The present study was designed to address this task.

Representatives of the family *Vibrionaceae* of gram-positive 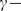
proteobacteria are among the most commonly reported bacteria to be found in extreme environments [7]. Currently, *Vibrionaceae* includes 130 highly diverse species divided into seven genera, including *Vibrio*, *Aliivibrio* and *Photobacterium* [11]. Representatives of this family populate almost all aquatic habitats, fresh water as well as sea or brackish waters, and it encloses psychrophilic, intermediate and mesophilic organisms. In the presented study we investigate membrane proteins present in the core-genome of 64 completely sequenced *Vibrionaceae* genomes, including sequences from three genera and 20 different species. The compilation of a dataset that comprises representatives of species from different genera is not free of criticism. Some studies limit the investigated sequences to proteins from closely related species to minimize phylogenetic effects in amino acid substition [12]. However, recent studies indicate that slow neutral substitution processes that are based on phylogenetic distance have little influence on the results of adaptation studies [13]. Additionally, a dataset that includes only genomes from relatively closely related organisms may not be able to identify general features of protein adaptation to low temperature. Thus, a set of genomes that belong to a relatively wide range of phyla can be more suitable for the investigation of general adaptation strategies. In addition to this, other factors are also reported to influence the outcome of these studies, such as varying GC-content or the comparison of bacteria from environmental niches that differ in more than one characteristic, e.g., optimal growth and hydrostatic pressure [14], [15]. In the presented study, we tried to overcome these problems by (i) compiling a diverse dataset from the bacterial family *Vibrionaceae* that enables the identification of general adaptation strategies and (ii) exclusively comparing transmembrane proteins that are part of the core-genome of our dataset, i.e., that are conserved in all investigated genomes.

## Results

### Classification of Psychrophilic, Intermediate and Mesophilic Organisms

The genomes in our dataset were divided into three distinct temperature groups: psychrophilic, intermediate and mesophilic. Table 1 summarizes the distribution of genomes from the different temperature groups in our dataset. In the presented study psychrophiles are defined as bacteria that are capable of growing at 4°C but not at 30°C. Furthermore, mesophilic organisms are defined as capable of growing at temperatures above 35°C. Additionally, an intermediate group was defined that includes bacteria that either (i) have a maximal growth temperature of 30°C but do not grow at 4°C or (ii) grow at 4°C but not above 35°C. Exact growth temperature ranges of 57 isolates were obtained from the literature [16]–[18] (see File S1 for details). Unfortunately, the exact growth temperature of the six remaining isolates could not be determined. However, five of these isolates were classified according to the general temperature groups, i.e., mesophilic or psychrophilic, as stated at the GOLD database [19]. Finally, based on the phylogenetic results from a recent study, the remaining *Vibrio sp.* MED222 was considered a representative of species*Vibrio splendidus* and was therefore classified accordingly [20]. In total the dataset used in the presented study includes four psychrophilic bacteria, six isolates assigned to the intermediate temperature group and 54 mesophilic bacteria.

**Table 1:** Genome dataset.

Organism	Genomes	Habitat		
		# psychrophilic	# intermediate	# mesophilic
***Aliivibrio***;				
*A. fischeri*	2	–	2	–
*A. salmonicida*	1	1	–	–
*A. wodanis*	1	1	–	–
***Vibrio***;				
*V. alginolyticus*	2	–	–	2
*V. anguillarum*	1	–	–	1
*V. campbellii*	1	–	–	1
*V. cholerae*	26	–	–	26
*V. coralliilyticus*	1	–	–	1
*V. furnissi*	1	–	–	1
*V. harveyi*	3	–	–	3
*V. metschnikovii*	1	–	–	1
*V. mimicus*	3	–	–	3
*V. orientalis*	1	–	1	–
*V. parahaemolyticus*	6	–	–	6
*V. splendidus*	2	–	2	–
*V. shilonii*	1	–	–	1
*V. vulnificus*	2	–	–	2
*V.* sp.	4	–	1	3
***Photobacterium***;				
*P. angustum*	1	–	–	–
*P. damselae*	1	–	–	–
*P. profundum*	2	2	–	–
*P* sp.	1	–	–	–

Summary of habitats populated by the genomes included in the study.

### Determination of Membrane Proteins

The set of core-genes of all 64 *Vibrionaceae* genomes was determined using the software orthoMCL [21] as described in detail in a previous study [20]. We applied the bioinformatic tools signalP [22], [23], HMMTOP [24], [25] and TMBETA-NET [26] to all core proteins and identified a set of 66 clusters of homologous membrane proteins present in all 64 investigated genomes. Furthermore, we used the software PSIPRED [27] for a more general secondary structure prediction of all identified membrane proteins. The prediction of specific types of membrane proteins resulted in a dataset of 52 transmembrane protein (TMP) families, 11 membrane protein families with a leading signal sequence, and 3 outer membrane proteins containing 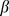-barrels. The group of 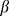-barrel membrane proteins was too small for a separate statistical analysis, but was included in the general analysis of all proteins. As shown in table 2, the homologous sequences of each cluster show a high degree of sequence identity within as well as between different temperature groups. This is not surprising as core-genes tend to be highly conserved, most notably for core genes of closely related organisms.

**Table 2:** Sequence similarity and sequence length.

	Mesophile	Intermediate	Psychrophile	Mean sequence length
**Mesophile**	0.6402–1.000			334.1
**Intermediate**	0.6529–0.8831	0.7070–0.9936		334.3
**Psychrophile**	0.6507–0.7796	0.6693–0.9123	0.6964–0.9750	334.4

Overall sequence similarity and mean sequence length within and between the three temperature groups of organisms. Protein length is given in # of amino acids.

### Amino Acid and Length Variations

**Figure 1:**
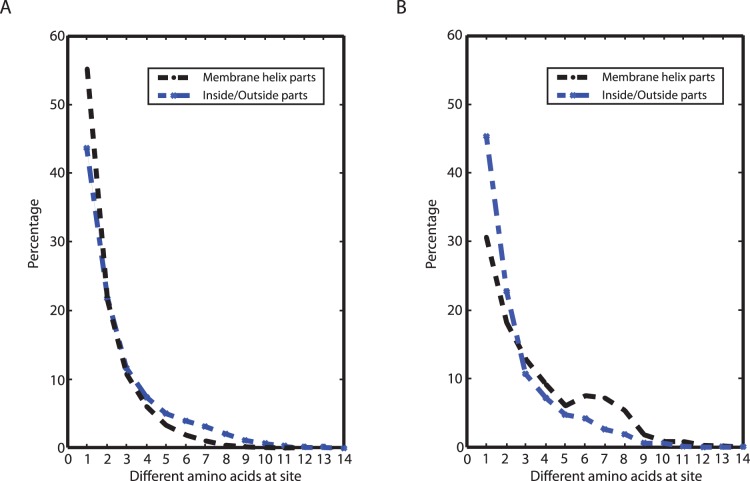
Distribution of the sites in the alignments according to their variability of amino acids. The data consists of 52 transmembrane proteins with 18024 sites altogether (A), and of 11 membrane proteins with signal peptides (B) enclosing 3248 sites. Both, the membrane part and the sequence part outside of the membrane, are shown in each plot. In the case where gaps occur at a site, this is counted as an extra amino acid. Note the similarity in distribution of the parts outside the membrane.

We calculated the length of transmembrane helices and, for signal peptides, of the signal sequence to investigate whether cold-adaptation affects the length of these sequence parts. Additionally, we investigated all helices as identified by PSIPRED to determine general changes in helix length of membrane proteins from mesophilic to psychrophilic temperature group. As shown in table 3 the length of all investigated sequence parts appeared to be similar for sequences in our dataset. Additionally, we examined the alignment site variability of amino acids within sequence parts located inside the membrane, i.e. transmembrane helices or signal sequences, and outside of the membrane. Figure 1A shows the results for the set of all 52 aligned TMPs. The site variations for the transmembrane helices range from 1 to 9 amino acids, with 55.27% of all sites fully conserved, and 0.0071% sites varying with 9 amino acids. For TMP sequence parts outside of a membrane the variability ranged from 1 to 13 amino acids, with 43.6% conserved sites, 1.04% with 9 amino acids and 0.0099% with 13 amino acids. Figure 1B shows the results of the same analysis for the 11 signal peptide alignments. For the sequence parts outside the membrane similar results as for the TMPs were achieved: variability ranged from 1 to 10, with 45.3% conserved sites, and 0.66% with 10 amino acids. However, amino acid variability for the signal sequences differed from the transmembrane proteins: variability ranged from 1 to 11 amino acids with 30.6% conserved among all alignment sites and 0.75% of the alignment cites varied in 10 and 11 amino acids, respectively. Furthermore, the amount of alignment sites that varied in 5 up to 8 amino acids was found to be increased in comparison to the transmembrane proteins.

**Table 3:** Mean length of signal sequences and helices.

	Signalsequence	TM helicesHMMTOP	All helicesPSIPRED
**Mesophile**	31.45	112.05	212.93
**Intermediate**	30.73	112.29	212.96
**Psychrophile**	28.43	113.17	212.45

Mean length (in # of aa) of the signal sequence of all 11 signal proteins, together with the total length of the helices embedded in the membrane (TM) as predicted by HMMTOP for the 52 transmembrane proteins. The length of all the helices as predicted by PSIPRED (both the membrane part and outside the membrane) in the proteins are added in the last column for comparison. All values are shown as mean values.

### Changes in Amino Acid Composition

**Figure 2:**
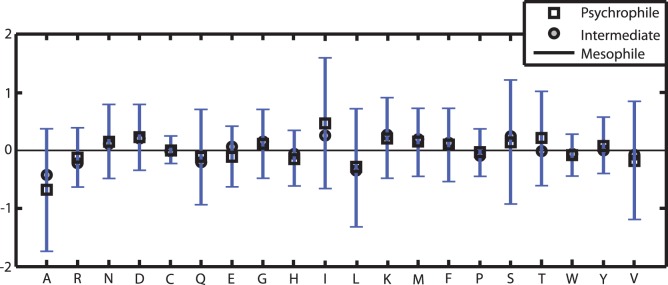
Comparison of the mean compositional differences in the 66 membrane core proteins. Changes are observed in the direction Mesophiles → Psychrophiles. Error bars represent the empirical standard deviations of the mesophile-psychrophile changes.

We analyzed the amino acid composition of each cluster of homologous membrane proteins to determine changes in the composition of psychrophilic sequences in comparison to their mesophilic counterparts. A paired Wilcoxon test for all 20 amino acids was performed separately on the group of TMPs and signal peptides as well as on the complete dataset. For each group compositional changes were calculated (i) for the entire sequence, (ii) sequence parts inside membranes and (iii) sequence parts outside membranes. Table 4 shows the results of the statistical analysis. Surprisingly, all changes in amino acid composition that were statistically significant (p-value <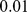) were found either for the complete sequence or for sequence parts outside of the membrane. For the transmembrane helices and signal sequences no significant preferences in amino acid composition were found in our dataset. The most central residues in terms of compositional changes are the amino acids Ala (A) and Ile (I) as they show significant differences in frequency for the complete sequences as well as for sequence parts outside the membrane in all investigated proteins. In the psychrophilic group A is significantly suppressed, while I is significantly enhanced (Figure 2). Additionally, the amino acids Lys (K), Asp (D) and Thr (T) are statistically increased for all 66 psychrophilic membrane proteins when compared to the mesophilic group. Furthermore, our results reveal that the amino acids Arg (R) and Glu (E) are underrepresented in the 52 psychrophilic TMP sequences.

**Table 4:** Differences in amino acid composition.

aa	Complete sequences		TMP			Signal peptides		
	All	Vibrio group	Tc	Tm	To	Sc	Sm	So
**A**	**<**10^−4^	.0012	**<**10^−4^	.02	.0002	.03	.24	.005
**R**	.06	.0007	.006	.72	.02	.10	.51	.07
**N**	.14	.12	.10	.05	.35	.90	.24	.52
**D**	.003	.0004	.004	.36	.004	.70	.95	.76
**C**	.90	.01	.87	.28	.02	.50	.30	1.0
**Q**	.18	.003	.35	.52	.74	.83	.49	.46
**E**	.08	.7	.009	.14	.009	.41	.84	.76
**G**	.13	.003	.14	.56	.13	.76	.28	.83
**H**	.05	.3	.05	.66	.11	.76	.91	.90
**I**	.0005	.07	.009	.78	.009	.003	.63	.007
**L**	.09	.0001	.04	.69	.03	1.0	.21	.64
**K**	.002	.002	.02	.63	.008	.28	.58	.17
**M**	.05	.002	.07	.31	.06	.17	.64	.28
**F**	.25	.01	.14	.04	.86	.90	.70	.90
**P**	.54	.001	.38	.24	.94	.37	.63	.90
**S**	.34	.02	.46	.92	.73	.63	.08	.21
**T**	.007	.8	.0003	.04	.009	.17	.12	.32
**W**	.12	.002	.17	.57	.37	.70	.44	.92
**Y**	.38	.2	.57	.67	.72	.46	.46	1.0
**V**	.25	.6	.71	.72	.19	.17	.21	.24

P-values from paired Wilcoxon tests of differences in amino acid composition. Shown are the results from the analysis of (i) the mesophilic and psychrophilic groups of the complete dataset (column 2 and 4–9) and (ii) the intermediate and mesophilic group of the genus *Vibrio* (column 3).**Complete sequences:** The results for all the 66 membrane protein clusters. *All:* analysis of the mesophilic and psychrophilic groups of the complete dataset. *Vibrio group:* results for the intermediate and mesophilic group of the genus *Vibrio*. **TMP:** Results of 52 transmembrane proteins (TMP) are shown in columns 4–6. *Tc*: complete sequence of all TMP cluster. *Tm*: membrane helices of TMPs. *To*: sequence parts of TMPs outside the membrane. **Signal peptides:** Results from all 11 signal peptide clusters are shown in column 7–9. *Sc*: complete sequence of all signal peptides. *Sm*: signal sequence of signal peptides (anchor). *So*: sequence parts outside membrane. Differences with p-values<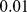 are shown in bold.

**Figure 3:**
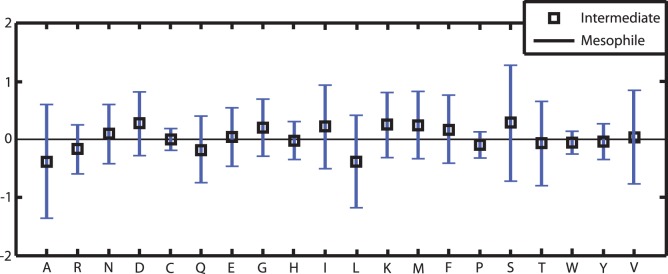
Comparison of the mean compositional differences in the 66 membrane core proteins in the sub-group of *Vibrio* genomes. Changes are observed in the direction mesophilic → intermediate group. Error bars represent the empirical standard deviations of the mesophile-intermediate changes.

Due to the fact that differing GC-content can have a significant effect on the compositional changes, we additionally investigated the changes in amino acid composition of a sub-group of genomes with close GC-content. Unfortunately, the two genera of our dataset that include psychrophilic organisms, *Aliivibrio* and *Photobacterium*, are represented by only four and five genomes, respectively. Furthermore, only the genus *Photobacterium* is represented by mesophilic as well as psychrophilic organisms. As the barophilic character of representatives of *Photobacterium profundum* may also have an effect on the amino acid composition we propose that it is unsuitable as a reference group to confirm our general findings. Therefore, we chose the group of 55 *Vibrio* genomes and determined whether the changes in amino acid composition between the groups of mesophilic and intermediate *Vibrio* genomes supports our general findings from the comparison of mesophilic and psychrophilic *Vibrionaceae*. Figure 3 and table 4 show that these results confirm some but not all findings from the complete dataset. Regarding the amino acid composition of Ala (A), Lys (K) and Asp (D) the results from the *Vibrio* sub-group are conform to those of the complete dataset. However, the comparison of the intermediate versus the mesophilic group of *Vibrio* genomes shows no statistically significant changes in the composition of amino acids Ile (I) and Thr (T) which was found when comparing all *Vibrionaceae*. Furthermore, we determined significant compositional changes for the *Vibrio* sub-group that were not found for all genomes. The amount of amino acids Gln (Q), Leu (L), Pro (P) and Trp (W) is significantly decreased in the amino acid sequence of the intermediate membrane proteins whereas the amino acids Asp (D), Gly (G) and Met (M) are significantly increased. Additionally, Arg (R) is also significantly decreased in the membrane proteins of the intermediate *Vibrio* group, which is not found for all proteins of the complete dataset but for the TMPs.

An additional analysis was performed to investigate preferences in amino acid substitutions between mesophilic and psychrophilic membrane proteins of the complete dataset. All possible 421 amino acid substitutions (gaps included) were analyzed for statistical bias by using the two-sided chi-square test. The results showed no statistically significant preferences (p-values <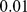) in the substitution pattern of amino acids (data not shown). Thus, our results show that, in terms of cold adaptation of membrane proteins, no single amino acid is primarily substituted by one specific amino acid.

### Physicochemical Properties

In order to determine physicochemical characteristics of psychrophilic membrane proteins we analyzed the physical, chemical and geometric properties of all 20 amino acids in the sequences of the cold-adapted temperature group in comparison to those of mesophiles. We chose several characteristics that are known to be important for cold-adaptation, mainly for psychrophilic enzymes [28], [29]. Table 5 shows the results including the statistical bias as well as specific trends for the transmembrane (TM) and the signal proteins (SP). In general, the homologous sequences of all clusters show very similar physicochemical properties. The only significant change for psychrophilic membrane proteins is the amount of hydrophobic amino acids. In comparison to the mesophilic group cold-adapted sequences show a significant decrease of hydrophobic amino acids in their primary protein structure. The reduced hydrophobicity is also observed by the general hydrophobicity scale [30] for the signal proteins (p-value = 0.04), and by the special hydrophobicity scale [31] for all membrane proteins (p-value = 0.14), as shown in table 6. Additional findings are that the isoelectric point of TMPs is decreased (p-value = 0.013), and molecular weight and the accessible surface area is increased in signal proteins.

**Table 5:** Changes in physicochemical properties.

AA group	Complete sequences	TMP			Signal peptides		
		Tc	Tm	To	Sc	Sm	So
**Hydrophobic**	↓ (.0008)	↓ (.004)	↓ (.15)	↓ (.05)	↓ (.07)	↓ (.08)	↓ (.12)
**Charged**	↑ (.94)	↓ (.23)	↓ (.31)	↓ (.63)	↑ (.15)	↓ (.27)	↑ (.17)
**Negative**	↑ (.30)	↑ (.80)	−(.64)	↑ (.69)	↑ (.27)	↑ (.96)	↑ (.63)
**Positive**	↑ (.55)	↓ (.67)	−(.86)	↓ (.72)	↑ (.71)	↓ (.12)	↑ (.07)
**Polar**	↑ (.12)	↑ (.05)	↑ (.12)	↑ (.30)	↑ (1.0)	↓ (.02)	↑ (1.0)

P-values from paired Wilcoxon tests of differences in amino acid composition between the mesophilic and psychrophilic groups. **Complete sequence**s: The results for all the 66 membrane protein cluster. Results of 52 transmembrane proteins (TMP) are shown in columns 3–5. **Tc**: complete sequence of all TMP cluster. **Tm**: membrane helices of TMPs. **To**: sequence parts of TMPs outside membrane. Results from all 11 signal peptide clusters are shown in column 6–8. **Sc**: complete sequence of all signal peptides. **Sm**: signal sequence of signal peptides (anchor). **So**: sequence parts outside membrane. Differences with p-values<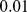 are shown in bold. Arrows indicate direction of change from the mesophilic to the psychrophilic group and are based on the median value of the observed changes.

**Table 6:** Changes in physicochemical peptide properties.

Property	Trend M → P	Trend M → P	Trend M → P
	All (p-value)	TMP (p-value)	SP (p-value)
# pos. charged aa	↑ (.42)	↓ (.69)	↑ (.07)
# neg. charged aa	↑ (.48)	↑ (.89)	↑ (.46)
# charged aa	↑ (.22)	↑ (.84)	↑ (.07)
Isoelectric point scale	↓ (.14)	↓ (.01)	↑ (.28)
# polar aa	↑ (.24)	↑ (.10)	↑ (.90)
Polarity scale	↑ (.93)	↓ (.21)	↑ (.08)
# hydrophobic aa	↓ (.003)	↓ (.03)	↓ (.03)
Hydrophobicity scale	↓ (.69)	↓ (.54)	↓ (.04)
Hydrophobicity membrane scale	↓ (.14)	↓ (.13)	↓ (.63)
Molecular weight	↑ (.10)	↑ (.32)	↑ (.03)
Accessible surface area	↑ (.09)	↑ (.37)	↑ (.02)
Bulkiness	↑ (.47)	↑ (.36)	↓ (.90)
Shape (position of branch point)	↓ (.18)	↓ (.06)	↑ (0.32)
Gibbs free energy change	↓ (.53)	↑ (.62)	↓ (.04)
Flexibility, peptide	↓ (.86)	↓ (.80)	↓ (.64)

Changes in number and physicochemical values of membrane proteins between the Mesophilic (M) and Psychrophilic (P) temperature groups. The results for all the 66 membrane protein families are shown in column 2, the 52 transmembrane proteins in column 3, and the 11 signal peptides in column 4. The p-values from the data adaptive paired t-test/Wilcoxon test (two-tailed) are included in parenthesis. The arrows indicate direction of change from the mesophilic to the psychrophilic group and are based on the median value of the observed changes.

As mentioned above, our results regarding compositional changes revealed a significant decrease in the frequency of the amino acid A in psychrophilic TMPs of our dataset. It has been shown that A is one of the best helix-forming residue in peptide sequences [32] and thus a decrease in A-content might increase protein flexibility due to a decreased ability of psychrophilic TMPs to form helices. To further investigate this hypothesis we calculated the secondary structure profiles of transmembrane proteins utilising the secondary structure propensity scale of all amino acids [33]. As shown in Table 7, these results confirmed a decrease of the helix forming property (p-value = 0.08 in the membrane parts, and p-value <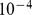 of the whole protein), indicating a lower degree of helix stabilization for psychrophilic TMPs in comparison to mesophilic sequences.

**Table 7:** Secondary structure propensities.

Region	Alpha	Beta	Loop
Entire sequence	↓ 10^−4^	↑ (.009)	↑ (.009)
Transmembrane part	↓ (.081)	–	–

Changes in secondary structure propensity from mesophile to psychrophile transmembrane proteins. The p-values from the data adaptive paired t-test/Wilcoxon test (two-tailed) are included in parenthesis. The arrows indicate direction of change from the mesophilic to the psychrophilic group and are based on the median value of the observed changes.

## Discussion

We performed a comparative bioinformatical analysis using protein alignments from the core-genome of 64 fully sequences *Vibrionaceae* genomes to determine general characteristics of cold-adaptation of membrane proteins, transmembrane proteins as well as signal peptides. We identified 66 membrane proteins, 52 TMPs, 11 signal peptides and 3 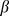-barrel containing membrane proteins, present in all genomes in our dataset. Analysis of a set of diverse membrane proteins that are part of the *Vibrionaceae* core-genome, rather than to focus on one particular protein or a phylogenetically narrow group of bacteria, increases the possibility that the characteristics found in this study are common for thermal adaptation of membrane proteins. Additionally, limiting our dataset to sequences conserved in all representatives of one bacterial family reduces the effects of phylogenetic variations on the presented results.

### Determined Variations of Signal Peptides

Signal peptides show common structural features, e.g., a positively charged N-terminal followed by a hydrophobic core and a more polar cleavage site [34], [35]. Variations in length of, e.g., the hydrophobic core of a signal sequence, can effect the accurate translocation or function of the mature peptide [36]. Furthermore, it has been shown that the interaction of the signal sequence with the hydrophobic lipid bilayer of the membrane is affected by environmental stress, e.g. varying temperature or salinity [37]. Therefore, it is legitimate to assume that signal sequences undergo a change in amino acid composition or length to adapt to low temperature. On the other hand, it has been shown that signal sequence do not always function efficiently when expressed in a different organism [38]. This indicates the host-specificity of at least a fraction of all signal sequences and thus changes in composition or length of signal sequences might be based on adaptation to the host and not to environmental conditions. Our results indicate that the signal sequence of psychrophiles differ from those of mesophilic bacteria in the level of conservation. However, as we were not able to determine a pattern in properties of the substituted amino acids or the exact location of the length difference, our results remain ambiguous in the context of cold adaptation strategies. The determined increase in site variability of the membrane parts of signal peptides may be caused by phylogenetic or functional differences of the sequences in our dataset and not based on adaptation to the cold. Another possible explanation is the size of the dataset as we included only 11 signal peptides. Future studies may address this problem by investigating single signal peptide families or larger datasets.

### General Features of Cold-adaptation in Membrane Proteins

The results from the analysis including the complete dataset, however, are more clear. The determined changes in amino acid composition are either found for the complete protein sequence or, more often, for sequence parts that are located outside of the membrane. As these sequence parts tend to be loop-like regions it is not surprising that our results resemble those reported for loop regions of cold-adapted enzymes. One key finding of the presented study is the increased amount of the amino acid I in the sequence parts of membrane proteins that are located outside of the lipid bilayer. Due to its hydrophobicity and helix-destabilizing property an increase in I decreases structural stability and thus increases the flexibility of loop regions in cold-adapted proteins [39]. Furthermore, a study recently published by Budde *et al.* on the gram-positive bacterium *Bacillus subtilis* showed the importance of membrane-associated I in the cold-shock response of *B. subtilis* [40], supporting the hypothesis that the increase in I is a common strategy in cold-adaptation of membrane associated proteins. As reported, the increase in I was not confirmed by the comparison of the intermediate versus the mesophilic group of *Vibrio* genomes. However, as shown in figure 2 the intermediate group of the complete dataset also shows an increase in I although less than in the psychrophilic group. We therefore propose that the general result regarding amino acid I was not confirmed by the *Vibrio* sub-group due to the fact that we did not compare psychrophiles but organisms of the intermediate group to mesophiles. Thus, we hypothesize that the increase of amino acid I would be significant for psychrophiles of the genus *Vibrio* as found for the complete dataset.

An additional finding is the decreased amount of the amino acid A in protein sequences of psychrophilic membrane proteins when compared to the mesophilic group. This result was confirmed by the comparison of the intermediate group versus the mesophiles of genus *Vibrio*. The lower amound of A in the peptide sequences decreases the stability of 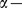
helices and thus the structural stability of the overal sequence, due to the fact that A is the best helix-forming residue [32]. The results of the calculated helix profiles supports this hypothesis as it reveals a decrease in helix forming properties for the investigated membrane sequences.

The remaining changes in amino acid composition show a clear preference for hydrophobic and helix-breaking or destabilizing amino acids. For example, the amino acid D has been reported to be helix-breaking [41] whereas T is helix indifferent. Also, the avoidance of amino acid E in psychrophilic TMPs can be explained by its trend to favor for helical structures and has also been reported for the proteomes of other psychrophilic organisms [39], [42]. Additionally, our results indicate that the amino acids K and R also play a role in adaptation of TMPs to low temperatures. Although the changes in frequency of these two amino acids lack an obvious interpretation, similar results have recently been reported for other psychrophilic bacteria, too [43], [44]. Additionally, these findings are also confirmed by our investigation of the sub-group of intermediate and mesophilic *Vibrio* genomes.

The differences in the results of the complete dataset in comparison to the sub-group of *Vibrio* genomes can be explained by multiple factors. First, psychrophilic representatives of different genera may show different strategies to adapt to low temperature. Therefore, the reported differences may be specific for the genus *Vibrio* and are not generally found for membrane proteins of the complete family of *Vibrionaceae*. Second, the group of *Vibrios* in our dataset does not include true psychrophiles but organisms that are representatives of the intermediate temperature group that are described as mostly *psychrotolerant*. Thus, the reported compositional changes might be specific to this temperature group and might not be found in psychrophiles of this genus. Although speculative, we favor this explanation, as the results of the intermediate *Vibrio* group show similarities to the intermediate group of the complete dataset in comparison to the mesophilic group of the complete dataset. Nevertheless, it has to be mentioned that the differences of the two analysis may also be based on GC-content and phylogenetic distance of the compared genomes and not on cold adaptation strategies.

The analysis of physicochemical properties of membrane proteins revealed a lower hydrophobicity in the psychrophilic sequences. This effect can be seen in Table 5 and 6, where it appears as a detectable indicator, and point out that a less hydrophobic protein is an important factor in cold adaptation of membrane proteins. The sequence regions of membrane proteins that are located outside of the membrane are exposed to water in either the cytoplasm or the periplasmic space. Low temperature affects the dynamics of water and water networks and decreases the ability to form hydrogen bonds. An increased hydrophobicity may compensate for these effects and was also recently reported for loop-regions of cold-adapted enzymes [8], [45].

In summary, our results show that cold-adaptation of membrane proteins almost exclusively effects parts of the proteins that are located outside of the membrane. For these sequence parts our analysis reveals similar cold-adaptation strategies as for loop regions of cold-adapted enzymes. This includes higher flexibility at the expense of stability, a decrease in the propensity to form helices as well as a decrease in hydrophobicity. Surprisingly, we were not able to identify characteristics of cold-adaptation of the sequence parts embedded in the membrane although low temperature has a strong effect on the lipid bilayer. Therefore, it might be reasonable to assume that psychrophilic organisms countervail the changes in membrane fluidity mostly by altering the composition of the membrane itself rather than to modify the transmembrane helix parts of its membrane proteins. Future studies including more sequences may confirm these results or give a deeper insights into the adaptation of membrane helices of psychrophilic bacteria.

## Materials and Methods

### Homolog Retrieval and the Comparative Analysis

We used the dataset of 64 *Vibrionacea* genomes, including 20 different species from the genera *Aliivibrio*, *Photobacterium* and *Vibrio*. The core genome was identified using the software orthoMCL. A detailed description of the dataset and the homology clustering can be found in Kahlke et al. 2012 [20].

Identification of putative signal and membrane proteins was performed by applying three different prediction tools to the complete set of core-genes of all 64 *Vibrionaceae* genomes. The transmembrane proteins and their transmembrane helices were predicted using HMMTOP v2.1 [24], [25]. These were used for statistical analysis of helix properties, e.g. length variations. Prediction of transmembrane 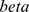-strands of outer membrane proteins was performed using TMBETA-NET [26] and signal peptides were identified using SignalP v3.0 [22], [23]. Additionally, the secondary structure of all identified transmembrane proteins was predicted using PSIPRED v3.21 [27].

All sequences of each homolog cluster were manually curated and those proteins were excluded that contained sequences with missing starts or stops, i.e. truncated sequences from draft genomes.

Finally, we compiled a dataset of 4224 protein sequences distributed over 66 cluster of predicted transmembrane sequences. The predicted sequences were conserved among all genomes included in our dataset. Paralogs were excluded from our analysis, hence each of the clusters contained 64 protein sequences. The sequences of each cluster of homologs were subsequently aligned using T-Coffee v.6.7 [46] and then divided into three different temperature groups based on the optimal growth temperature or normal living condition for the species: psychrophile (4 sequences), intermediate (6 sequences) and mesophile (54 sequences). Table 1 shows a summary of our dataset.

### Changes in Gene Composition and Properties

We have applied the new methods of comparative statistical bioinformatics as described in a recent paper by Thorvaldsen et al. (2010) [47], and amino acid composition and substitution were examined by the toolbox DeltaProt [47]. Since there are several sequences of the same species in each temperature group, each temperature group was treated as a statistically dependent sample of data, and hence the analysis used statistical tests for dependent data (Thorvaldsen et al 2010, table 2). The statistical tests were applied on the mean values of each group and used as representative observations. Our data set is unbalanced since there is different number of sequences in each temperature group, and the variances of each of the mean values will be unequal. Therefore, we used a non-parametric paired Wilcoxon sign-rank test as the appropriate test since it only assumes that the mean values have symmetrical shapes and a common mean. The pairing is based on equal (homologous) proteins from the two temperature groups, i.e., mesophiles and psychrophiles. By this we examine the change in composition of amino acids in the different temperature groups. We also looked for amino acid directional biases in substitutions among the proteins from the different groups, and for general change in physicochemical properties of the involved amino acids. We applied the same physicochemical properties as in Thorvaldsen et al. (2007, 2009) [48], [49].

## Supporting Information

File S1 **Species name, strain and temperature habitat of the studied bacteria.**
(DOC)
