# Supplementary material for: Molecular Characterization of Cold Adaptation of Membrane Proteins in the Vibrionaceae Core-Genome
Source: PLoS One. 2012 Dec 17;7(12):e51761. doi: 10.1371/journal.pone.0051761 (PMC3524096; doi:10.1371/journal.pone.0051761)
Supplement: File S1 — Species name, strain and temperature habitat of the studied bacteria. (DOC) [file pone.0051761.s001.doc]

| **Organism** | **Habitat*** | **T_opt_** | **T_min_** | **T_max_** | **GC content (%)** | **Genbank accession numbers** |
| --- | --- | --- | --- | --- | --- | --- |
| *A. fischeri* str. ES114 | I | 20-28°C(**^3)^** | > 4°C(**^1)^** | 30°C(**^1)^** | 38 | CP000020  CP000021  CP000022 |
| *A. fischeri* str. MJ11 | I | 20-28°C(**^3)^** | > 4°C(**^1)^** | 30°C(**^1)^** | 38 | CP001133  CP001139  CP001134 |
| *A. salmonicida* str. LFI1238 | P | 15°C**^(3)^** | 1°C**^(2)^** | < 25°C**^(2)^** | 38 | FM178379  FM178380  FM178381  FM178382  FM178383  FM178384 |
| *A. wodanis* str. 06/09/139 **^+^** | P | NA | 4°C **^(1)^** | < 30°C **^(1)^** | 39 | NA |
| *V. alginolyticus* str. 12G01 | M | NA | > 4°C **^(1)^** | 40°C **^(1)^** | 44 | AAPS00000000 |
| *V. alginolyticus* str. 40B | M | NA | > 4°C **^(1)^** | 40°C **^(1)^** | 44 | ACZB00000000 |
| *V. anguillarum* str. NB10 **^+^** | M | NA | > 4°C **^(1)^** | 35°C **^(1)^** | 44 | NA |
| *V. campbellii* str. AND4 | M | 30-35°C(**^3)^** | > 4°C **^(1)^** | 35°C(**^1)^** | 44 | ABGR00000000 |
| *V. cholerae* str. 12129-1 | M | NA | > 4°C **^(1)^** | 40°C **^(1)^** | 47 | ACFQ00000000 |
| *V. cholerae* str. 1587 | M | NA | > 4°C **^(1)^** | 40°C **^(1)^** | 47 | AAUR00000000 |
| *V. cholerae* str. 2740-80 | M | NA | > 4°C **^(1)^** | 40°C **^(1)^** | 47 | AAUT00000000 |
| *V. cholerae* str. 623-39 | M | NA | > 4°C **^(1)^** | 40°C **^(1)^** | 47 | AAWG00000000 |
| *V. cholerae* str. AM-19226 | M | NA | > 4°C **^(1)^** | 40°C **^(1)^** | 47 | AATY00000000 |
| *V. cholerae* str. B33 | M | NA | > 4°C **^(1)^** | 40°C **^(1)^** | 47 | AAWE00000000 |
| *V. cholerae* str. biovar albensis VL426 | M | NA | > 4°C **^(1)^** | 40°C **^(1)^** | 47 | ACHV00000000 |
| *V. cholerae* str. BX 330268 | M | NA | > 4°C **^(1)^** | 40°C **^(1)^** | 47 | ACIA00000000 |
| *V. cholerae* str. CIRS 101 | M | NA | > 4°C **^(1)^** | 40°C **^(1)^** | 47 | ACVW00000000 |
| *V. cholerae* str. CT5369-39 | M | NA | > 4°C **^(1)^** | 40°C **^(1)^** | 47 | ADAL00000000 |
| *V. cholerae* str. INDRE 91-1 | M | NA | > 4°C **^(1)^** | 40°C **^(1)^** | 47 | ADAK00000000 |
| *V. cholerae* str. M66-2 | M | NA | > 4°C **^(1)^** | 40°C **^(1)^** | 47 | CP001233  CP001234 |
| *V. cholerae* str. MAK 757 | M | NA | > 4°C **^(1)^** | 40°C **^(1)^** | 47 | AAUS00000000 |
| *V. cholerae* str. MJ1236 | M | NA | > 4°C **^(1)^** | 40°C **^(1)^** | 47 | CP001485  CP001486 |
| *V. cholerae* str. MO10 | M | 20-30°C(**^3)^** | > 4°C **^(1)^** | 40°C **^(1)^** | 47 | AAKF00000000 |
| *V. cholerae* str. MZO-2 | M | NA | > 4°C **^(1)^** | 40°C **^(1)^** | 47 | AAWF00000000 |
| *V. cholerae* str. MZO-3 | M | NA | > 4°C **^(1)^** | 40°C **^(1)^** | 47 | AAUU00000000 |
| *V. cholerae* str. NCTC 8457 | M | NA | > 4°C **^(1)^** | 40°C **^(1)^** | 47 | AAWD00000000 |
| *V. cholerae O1 biovar El Tor* str. N16961 | M | 20-30°C(**^3)^** | > 4°C **^(1)^** | 40°C **^(1)^** | 47 | AE003852  AE003853 |
| *V. cholerae* str. O395 | M | 20-30°C(**^3)^** | > 4°C **^(1)^** | 40°C **^(1)^** | 47 | CP000626  CP000627 |
| *V. cholerae* str. RC27 | M | NA | > 4°C **^(1)^** | 40°C **^(1)^** | 47 | ADAI00000000 |
| *V. cholerae* str. RC385 | M | 20-30°C(**^3)^** | > 4°C **^(1)^** | 40°C **^(1)^** | 47 | AAKH00000000 |
| *V. cholerae* str. RC9 | M | NA | > 4°C **^(1)^** | 40°C **^(1)^** | 47 | ACHX00000000 |
| *V. cholerae* str. TMA 21 | M | NA | > 4°C **^(1)^** | 40°C **^(1)^** | 47 | ACHY00000000 |
| *V. cholerae* str. V51 | M | 20-30°C(**^3)^** | > 4°C **^(1)^** | 40°C **^(1)^** | 47 | AAKI00000000 |
| *V. cholerae* str. V52 | M | 20-30°C(**^3)^** | > 4°C **^(1)^** | 40°C **^(1)^** | 47 | AAKJ00000000 |
| *V. coralliilyticus* str. ATCC BAA-450 | M**^(3)^** | NA | NA | NA | 45 | ACZN00000000 |
| *V. furnissi* str. CIP-102971 | M | 20-30°C(**^3)^** | > 4°C **^(1)^** | 40°C **^(1)^** | 50 | ACZP00000000 |
| *V. harveyi* str. 1DA3 | M | NA | > 4°C **^(1)^** | 35°C **^(1)^** | 45 | ACZC00000000 |
| *V. harveyi* str. ATCC BAA-1116 | M | 20-30°C(**^3)^** | > 4°C **^(1)^** | 35°C **^(1)^** | 45 | CP000789  CP000790  CP000791 |
| *V. harveyi* str. HY01 | M | NA | > 4°C **^(1)^** | 35°C **^(1)^** | 45 | AAWP00000000 |
| *V. metschnikovii* str. CIP 69-14 | M | NA | > 4°C **^(1)^** | 40°C **^(1)^** | 44 | ACZO00000000 |
| *V. mimicus* str. VM223 | M | NA | 4°C **^(6)^** | 40°C **^(6)^** | 46 | ADAJ00000000 |
| *V. mimicus* str. VM573 | M | 20-30°C(**^3)^** | 4°C **^(6)^** | 40°C **^(6)^** | 46 | ACYV00000000 |
| *V. mimicus* str. VM603 | M | NA | 4°C **^(6)^** | 40°C **^(6)^** | 46 | ACYU00000000 |
| *V. orientalis* str. CIP 102891 | I | NA | 4°C **^(1)^** | 35°C **^(1)^** | 44 | ACZV00000000 |
| *V. parahaemolyticus* str. 16 | M | 20-30°C(**^3)^** | > 4°C **^(1)^** | 40°C **^(1)^** | 46 | ACCV00000000 |
| *V. parahaemolyticus* str. AQ3810 | M | NA | > 4°C **^(1)^** | 40°C **^(1)^** | 45 | AAWQ00000000 |
| *V. parahaemolyticus* str. AQ4037 | M | NA | > 4°C **^(1)^** | 40°C **^(1)^** | 45 | ACFN00000000 |
| *V. parahaemolyticus* str. K5030 | M | NA | > 4°C **^(1)^** | 40°C **^(1)^** | 45 | ACKB00000000 |
| *V. parahaemolyticus* str. Peru-466 | M | NA | > 4°C **^(1)^** | 40°C **^(1)^** | 45 | ACFM00000000 |
| *V. parahaemolyticus* str. RIMD 2210633 | M | 20-30°C(**^3)^** | > 4°C **^(1)^** | 40°C **^(1)^** | 45 | BA000031  BA000032 |
| *V. shilonii* str. AK1 | M**^(3)^** | NA | NA | NA | 43 | ABCH00000000 |
| *V. sp.* EX25 | M**^(3)^** | NA | NA | NA | 44 | AAKK00000000 |
| *V. splendidus* str. 12B01 | I | NA | > 4°C **^(1)^** | < 30°C **^(1)^** | 44 | AAMR00000000 |
| *V. splendidus* str. LGP32 | I | NA | > 4°C **^(1)^** | < 30°C **^(1)^** | 44 | FM954972  FM954973 |
| *V. sp.* MED222 | I | NA | > 4°C **^(1,4)^** | < 30°C **^(1,4)^** | 43 | AAND00000000 |
| *V. sp.* RC341 | M**^(3)^** | 20-30°C(**^3)^** | NA | NA | 46 | ACZT00000000 |
| *V. sp.* RC586 | M**^(3)^** | 20-30°C(**^3)^** | NA | NA | 46 | ADBD00000000 |
| *V. vulnificus* str. YJ016 | M | 20-30°C(**^3)^** | > 4°C **^(1)^** | 40°C **^(1)^** | 46 | BA000037  BA000038  AP005352 |
| *V. vulnificus* str. CMCP6 | M | 20-30°C(**^3)^** | > 4°C **^(1)^** | 40°C **^(1)^** | 46 | AE016795  AE016796 |
| *P. angustum* str. S14 | M | 25°C **^(5)^** | 4°C **^(6)^** | 37°C **^(6)^** | 39 | AAOJ00000000 |
| *P. damselae* str. CIP 102761 | M | 26°C **^(6)^** | > 4°C **^(6)^** | 35°C **^(6)^** | 41 | ADBS00000000 |
| *P. profundum* str. 3tck | P | 15°C(**^6)^** | 4°C **^(6)^** | 18°C **^(6)^** | 41 | AAPH00000000 |
| *P. profundum* str. SS9 | P | 15°C(**^6)^** | 4°C **^(6)^** | 18°C **^(6)^** | 41 | CR354531  CR354532  CR377818 |
| *P. sp.* SKA34 | M**^(3)^** | NA | NA | NA | 39 | AAOU00000000 |

**Additonal file 1:**  Species name, strain and temperature habitat of the studied bacteria.

(*) M: Mesophilic, I: Intermediate; P: Psychrophilic.

(+) CDS blast server of unpublished sequences is available under <http://arctic.imb.fm.uit.no/blast/blast.html>

(1) Garrity, G (2005),  *Bergey’s Manual of Systematic Bacteriology*. Plenum US, 2005, Vol 2B, 524-527 and 549

(2) Egidius, E., Wiik, R., Andersen, 301 K., Hoff, K. A. & Hjeltnes, B. (1986). *Vibrio salmonicida* sp. nov., a new fish pathogen. *Int J Syst Bacteriol* 36, 518-520

(3) Taken from Genomes Online Database ([www.genomesonline.org](http://www.genomesonline.org/))

(4) Kahlke T, Goesmann A, Hjerde E, Willassen NP, Haugen P (2012) Unique core genomes of the bacterial family vibrionaceae: Insights into niche adaptation and speciation.

BMC Genomics 13:179

(5) Nogi, Y., Masui, N. & Kato, C. (1998c). *Photobacterium profundum* sp. nov., a new, moderately barophilic bacterial species isolated from a deep-sea sediment.

Extremophiles 2, 1-7.

(6) Stoica Costin, 2007-2012. *Regnum Prokaryotae*, http://www.tgw1916.net, accessed on October 2012
